# The role of laparoscopy in suspicious abdomen pain in children

**Published:** 2013

**Authors:** Zekeriya Ilce, Turan Yildiz, Mustafa Isleyen

**Affiliations:** 1 Dr. Zekeriya Ilce, Associate Professor, Department of Pediatric Surgery, Faculty of Medicine, Sakarya University, Sakarya, Turkey.; 2 Dr. Turan Yildiz, Assistant Professor, Department of Pediatric Surgery, Faculty of Medicine, Sakarya University, Sakarya, Turkey.; 3 Mustafa Isleyen, MD, Radiology Clinic, Elbistan Public Hospital, Kahmamanmaras, Turkey.

**Keywords:** Children, Laparascopy, Suspicious abdominal pain

## Abstract

***Objective:*** Abdominal pain is a frequently encountered problem in children. Suspicious right lower quadrant pains are sometimes a problem for pediatric surgeons. In this study, we wanted to evaluate the effectiveness of laparoscopy in diagnosis and treatment of children with suspicion of abdominal pain.

***Methods:*** The files of 84 patients treated with a diagnosis of suspicious right lower quadrant peritonitis between 2005 and 2011 were investigated. Laparoscopic exploration was performed for all cases with right lower quadrant pain where the appendix was not seen on USG/CT and the cause of acute abdomen could not be determined.

***Results: ***In this process, 84 patients consisting of 60 (71%) females and 24 (29%) males were included in the study. The mean age was 10.5 years (7-16 years). Appendicitis was determined during diagnostic laparoscopy in 35 (41.6%) patients. The appendix was normal in the remaining 49 (58.3%) patients. In 36 of these patients, gynecological diseases were encountered on pathology as the most frequent cause of the acute abdomen. The appendix was preserved in patients where the cause of the abdominal pain was explained.

***Conclusion: ***Laparoscopy can be used in the diagnosis and treatment of patients with suspected acute abdomen that imitates acute appendicitis and cannot be differentiated with physical examination and laboratory methods. Delays in diagnosis and unnecessary appendectomy will be prevented in this way.

## INTRODUCTION

Suspected acute abdomen is a common problem in children.^1^^-^^3^ Acute appendicitis has been diagnosed in 2.3% of children presenting at emergency, pediatric, and pediatric surgery clinics with abdominal pain.^4^ Acute appendicitis is the most common cause of surgical abdominal pain in children.^5^ Despite the increase in information about appendicitis, the rate of accurate diagnosis is still insufficient. Early diagnosis of acute appendicitis in infants and children reduces the rates of perforation and postoperative complications and shortens the duration of hospitalization.^6^^,^^7^

An atypical clinical picture is present in 1/3 of children with appendicitis, making diagnosis difficult. Obesity, gynecological pathologies in the adolescent period and communication difficulties with small children also present the surgeon with a dilemma. As a result, perforation was found in 23-73% of the cases who were operated on for appendicitis and the appendix was found to be normal in 15-25%.^8^ A noticeable increase was seen in mortality and morbidity rates after negative appendectomy.^9^ The complication rate following negative appendectomy was 11%.^10^ Today, the use of the appendix more frequently for appendicovesicostomy (Mitrofanoff) and antegrade continence enema (ACE) procedures has increased its importance, making more careful consideration of appendectomy necessary.^11^^,^^12^

The use of laparoscopy for abdominal exploration and treatment in suspected acute abdominal cases has increased in popularity in recent years. However, diagnostic laparoscopy is an invasive method and there have been discussions regarding its application on all patients diagnosed with or suspected of appendicitis.^13^^-^^16^

This study evaluates the effectiveness of laparoscopy in diagnosis and treatment of children with suspicion abdominal pain. We also share our experiences of suspicions abdominal pain and laparascopic appendectomy in children.

## METHODS

A total of 84 consecutive patients with suspected right lower quadrant peritonitis were operated between September 2005 and 2011 at the pediatric surgery department. Typical appendicitis cases were not included in the study. Parents’ informed consents were obtained before the surgery.

The surgeon decided to perform laparoscopic exploration on areas with sensitivity for all cases with right lower quadrant pain, the appendix could not be visualized on USG/CT, and the cause of acute abdominal pain remained unknown. These patients revealed minimal free fluids or no pathologies in US/CT. The data, clinical history, physical examination, vital signs, white blood cell count, C-reactive protein (CRP), urinalysis, chest and abdominal x-ray, ultrasonography (USG), computed tomography (CT) (some patients), intraoperative and postoperative complications, reasons for conversion to open surgery, and postoperative results of these patients were evaluated and entered into the database for analysis.

Local ethical committee approval was obtained for the study (Sakarya University Ethical Committee no: 71522473.050.01.04/40).

***Laparoscopic procedure: ***Laparoscopic surgery was performed under general anesthesia. The patient was placed on the operating table with the surgeon on the patient’s left. Preoperative nasogastric tube and urinary catheters were inserted in all patients. Pneumoperitoneum was created with a Veres needle or open technique through the umbilicus. Subsequently, a 10-mm trocar was placed from umbilicus. It was inserted with a 0^o^ camera. A 5-mm trocar was inserted from the left lower quadrant and supra-pubic area. After exploration of the abdominal cavity, other trocars were inserted in consideration of the pathology. The patient underwent appendectomy in the presence of appendicitis (appendix edematous/hyperemic/ erectile/phlegmonous, etc.) or in the absence of no other pathologies explaining the lower quadrant peritonitis. The appendix stump was sutured extracorporeally with a polyglycolic acid knot. The appendix was left in place and intervention for the pathology was performed if the appendix was normal and peritonitis could be explained. All removed materials were sent to laboratory for histopathological diagnosis.

## RESULTS

A total of 84 patients (60 female, 24 male) underwent laparoscopic surgery during this period. The mean age was 10.6±2.8 years, range 7-16 years. Appendicitis was found during laparoscopy in 35 patients. There was no pathology to explain the underlying cause of abdominal pain in 13 patients. Thus, 48 patients (57.1%) underwent appendectomy during laparoscopy. The diagnoses were histopathologically confirmed. In this series, the preoperative diagnosis of 49 patients (58.3%) was changed by laparoscopy and the appendices of these patients appeared normal. The underlying cause was determined and the appendix was left in place in 36 patients (9 male, 27 female). Gynecological diseases were found in 17 patients (8 ovarian cyst ruptures, 5 ovarian cysts, 2 ovarian cyst torsions, 1 ovarian abscess, 1 duplicate fallopian tube necrosis). Mesenteric lymphadenopathy, omentum torsion, Meckel's diverticulitis, and primary peritonitis were seen in 19 male and female patients. The appendix was preserved in these patients (42.8%). 

The distribution of the patients is shown in Table-I. The body weight of 15 of 84 patients was above the 90th percentile. Of these obese patients, we found omentum torsion in 4, appendicitis in 3, ovarian cyst in 2, ovarian cyst rupture in 2 and non-specific abdominal pain in 4. We converted to open surgery in 5 (5.9%) of the patients who underwent laparoscopy. In gynecological patients, we aimed to protect the ovary and adjacent organs (ovarian abscess) and determine the relationship between the fallopian tube and anomaly (duplicate fallopian tube necrosis). In addition, we converted to open surgery in 2 patients with retrocecal appendicitis and one patient with Meckel's diverticulitis due to bleeding during laparoscopy. No acute abdomen was determined in thirteen patients and they underwent appendectomy. The histopathological results of these patients were also found to be normal.

**Table-I:** The conditions of patients’ appendices and diagnoses

*Diagnosis*	*n* *(84)*	*Female* *(60)*	*Male* *(24)*	*Condition of appendix*
Appendicitis	35	23	12	33 Laparoscopic appendectomy and 2 converted open surgery were performed
Mesenteric lymphadenopathy	13	9	4	Appendix left in place
Ruptured ovarian cyst	8	8	-	Appendix left in place
Ovarian cyst	5	5	-	Appendix left in place
Torsioned ovarian cyst	2	2	-	Appendix left in place
Torsed mesentery	4	1	3	Appendix left in place
Ovarian abscess	1	1	-	Appendix left in place
Duplicated fallopian tube torsion	1	1	-	Appendix left in place
Meckel diverticulitis	1	-	1	Appendix left in place
Primary peritonitis	1	1	-	Appendix left in place
Non-specific abdominal pain	13	9	4	Laparoscopic appendectomy was performed

The rate of preoperative diagnostic change was 61.6% in adolescent females and 54.1% in adolescent males. The laparoscopic treatment rate was 94.04%. The preoperative clinical follow-up duration of the patients was 35.7±11.9 (12-60) hours and the length of hospital stay was 3.5±0.8 (2-6) days. The mean follow-up duration was 4 (2-6) months. The appendix stump was opened postoperatively in one patient in this study and the stump was repaired by laparotomy 3 days after the first operation. There were no postoperative mortalities.

## DISCUSSION

Acute appendicitis is the most common surgical emergency in children. Early diagnosis hinders the development of perforation, prevents late complications such as adhesion and abscess, and reduces morbidity and mortality in children.^17^ It can sometimes be difficult to make a diagnosis with non-invasive techniques. Unfortunately, appendectomy is performed although the appendix is normal in 15-25% of the patients who present with right lower quadrant pain and suspected acute abdomen.^8^ The correct preoperative diagnosis is therefore very important to reduce morbidity and mortality. Many techniques are used to reduce the negative appendectomy rates. These include close observation, laboratory tests, ultrasonography, computed tomography and even peritoneal cytology. Close observation and especially the imaging techniques of USG and computed tomography (CT) are widely used in the diagnosis of appendicitis in children^18^.

However, the benefits may be limited in the diagnosis of adolescent girls, atypical cases and obese children. Various studies report the sensitivity of CT in acute appendicitis as 87% and it is also reported to reduce the negative appendectomy rates from 14% to 4%. However, the routine use is controversial due to radiation during CT, especially in children.^18^^-^^20^ USG may be more useful in children than in adults because of the low amount of subcutaneous adipose tissue. However, its benefit in the diagnosis of obese children may be limited.^21^^,^^22^ Gynecological pathologies in adolescent girls can be confused with acute appendicitis, and this situation causes delays in diagnosis.^23^

Laparoscopy is used in the diagnosis and treatment of various emergency conditions (such as appendicitis, pelvic diseases, and colonic perforation).^24^ It is also highly sensitive in detecting abdominal pathology and can be used successfully in the treatment. Studies show that diagnostic laparoscopy reduces the number of negative laparotomies in suspected appendicitis and prevents severe peritonitis that may occur as a result of delays in diagnosis.^13^^,^^14^^,^^25^

In this study, the surgeon was left with a dilemma regarding definitive diagnosis following physical examination, laboratory tests, imaging methods, and clinical follow-up in 84 patients. A decision to perform diagnostic laparoscopy was therefore made. We were able to prevent unnecessary appendectomy and provide a proper diagnosis and treatment in 36 (42.8%) of the 84 patients as a result of laparoscopy. We also prevented morbidity due to negative appendectomy and preserved the appendix for possible use for another purpose later on in the child's life (Mitrofanoff, ACE procedure). On the other hand, laparoscopy is an invasive method and requires general anesthesia. Routine use of laparoscopy in appendicitis or suspected appendicitis cases in children is still controversial.

The routine use of laparoscopy may sometimes have more harm than benefits with the increased cost and hardware requirements, long surgical time and its own morbidity.^26^^-^^28^ However, the use of appendectomy when patients who have undergone laparoscopy because of right lower quadrant pain have an appendix with normal appearance and no other intra-abdominal pathology is still controversial.^29^ Based on the data, we preferred to perform appendectomy on 15.4% of the patients with a normal-appearing appendix where we encountered no other intra-abdominal pathologies. We believe that laparoscopy is much more valuable in the diagnosis and treatment of patients with clinically suspected appendicitis that leave the surgeon in a dilemma.

In addition, we believe that we may benefit from some major advantages by using the technique of laparoscopy in patients with suspected appendicitis, including an increase in the rate of correct diagnosis, preventing definitive operation delay, preservation of the appendix that can currently be used in different operations, and finally the use of laparoscopy for treatment. The majority of the patients (except for the non-specific 13 patients) had accurate diagnoses by laparoscopy. No significant difference was seen in the rate of changed preoperative diagnosis following laparoscopy (61.6%/50%) between male and female patients. Laparoscopy was helpful in diagnosing gynecological diseases in adolescent girls and obese children, preventing unnecessary laparotomy.

## CONCLUSION

Laparoscopy can be recommended in the diagnosis and treatment of cases who have suspected right lower quadrant peritonitis that cannot be diagnosed by physical examination and non-invasive methods, and that simulates acute appendicitis. We think that morbidity and mortality can be reduced by preventing unnecessary appendectomy and diagnostic delays. However, further studies are needed on the necessity of incidental appendectomy in nonspecific abdominal pain, especially in children.

## Notes

***Conflict and interests: ***The authors do not have any financial relationship with the organization that sponsored the research.
